# Changes in the vascular cell adhesion molecule-1, intercellular adhesion molecule-1 and c-reactive protein following administration of aqueous extract of *piper sarmentosum *on experimental rabbits fed with cholesterol diet

**DOI:** 10.1186/1476-511X-10-2

**Published:** 2011-01-09

**Authors:** Adel A Amran, Zaiton Zakaria, Faizah Othman, Srijit Das, Hesham M Al-Mekhlafi, Nor-Anita MM Nordin

**Affiliations:** 1 Department of Physiology, Faculty of Medicine, Universiti Kebangsaan Malaysia, Jalan Raja Muda Abdul Aziz, Kuala Lumpur 50300, Malaysia; 2 Department of Anatomy, Faculty of Medicine, Universiti Kebangsaan Malaysia, Jalan Raja Muda Abdul Aziz, Kuala Lumpur 50300, Malaysia; 3 Department of Parasitology, Faculty of Medicine, University of Malaya, Kuala Lumpur 50603, Malaysia

## Abstract

**Background:**

Inflammation process plays an important role in the development of atherosclerosis. Hypercholesterolemia is one of the major risk factors for atherosclerosis. The present study aimed to evaluate the effect of aqueous extract of *Piper sarmentosum (P.s) *on inflammatory markers like vascular cell adhesion molecule-1 (VCAM-1), intercellular adhesion molecule-1 (ICAM-1), and C-reactive protein (CRP).

**Methods:**

Forty two male New Zealand white rabbits were divided equally into seven groups; (i) C- control group fed normal rabbit chow (ii) CH- cholesterol diet (1%cholesterol) (iii) X1- 1% cholesterol with water extract of *P.s *(62.5 mg/kg) (iv) X2- 1% cholesterol with water extract of *P.s *(125 mg/kg (v) X3- 1% cholesterol with water extract of *P.s *(250 mg/kg) (vi) X4- 1% cholesterol with water extract of *P.s *(500 mg/kg) and (vii) SMV group fed with 1% cholesterol supplemented with simvistatin drug (1.2 mg/kg). All animals were treated for 10 weeks. Blood serum was taken for observing the inflammatory markers at the beginning and end of the experiment.

**Results:**

Rabbits fed with 1% cholesterol diet (CH) showed significant increase in the level of VCAM-1, ICAM-1 and CRP compared to the C group. The levels of VCAM-1, ICAM-1 and CRP in the 1% cholesterol group and supplemented with *P.s *(500 mg/kg) were significantly reduced compared to the cholesterol group. Similar results were also reported with simvistatin group.

**Conclusion:**

These results suggest that the supplementation of *Piper sarmentosum *extract could inhibit inflammatory markers which in turn could prevent atherosclerosis.

## Background

Inflammation is a major pathophysiological mechanism in atherosclerosis [1]. Histopathologically, atherosclerosis is characterized by a thickening of the vascular wall due to lipid accumulation and infiltration of macrophages and lymphocytes [2,3]. Many evidences have indicated the presence of many cell adhesion molecules and growth factors in atherosclerosis. Other reports have stressed that there are interactions between the adhesion molecules and growth factors in inflammatory responses. Researches have shown that the development of atherosclerosis is caused by a complex interaction between reactive oxygen species, lipids, endothelium, circulation and tissue inflammatory cells, platelets and vascular smooth muscle cells and was not simply due to the accumulation of lipids [4,5].

Hypercholesterolemia is one of the most important risk factors for atherosclerosis and related occlusive vascular disease [6]. Recent observations suggest that the endothelium's dysfunction and inflammation cause not only the initial stage of the atherosclerotic process but also atherosclerotic plaque development.

Vascular cell adhesion molecule VCAM-1 and intercellular adhesion molecule ICAM-1 are endothelial adhesion molecules of the Ig gene superfamily that may participate in atherogenesis by promoting monocyte accumulation in the arterial intima [7]. Elevated levels of inflammatory markers, especially CRP, are associated with an increased risk of cardiovascular disease [1,8,9]. Serum CRP stimulates endothelial cell expression of (ICAM-1) and (VCAM-1) [10]. The ICAM-1 and VCAM-1 are important factors in the development of atherosclerosis and may play an important role in promoting the local inflammation within the atherosclerotic plaque [11]. Cellular adhesion molecules like ICAM-1 and VCAM-1 are involved in the rolling, adhesion and extravasation of monocytes and T-lymphocytes [12-14]. This migration is one of the earliest events in the atherosclerotic process in addition to its later contribution to the chronic inflammatory process. Pathological studies of human atherosclerosis have shown increased expression of CAMs located in endothelial cells. Increased levels of soluble (s) forms of CAMs in circulating blood have been found in a number of conditions with an inflammatory component [15,16]. Various studies have showed there are many inflammatory markers that can predict cardiovascular events, including cell adhesion molecules, cytokines, chemokines, acute phase reactants such as fibrinogen, serum amyloid A and CRP. Additionally, CRP which has recently emerged as one of the most important inflammatory mediators can directly participate in the pathogenesis of atherosclerosis by activating endothelial cells and promoting the inflammatory component of atherosclerosis [2,3]. CRP plays a crucial role in the pathogenesis of the vascular inflammatory process which is abrogated by simvastatin therapy. In this study we focus on the effect of natural antioxidant on inflammatory markers, ICAM-1, VCAM-1 and CRP. The results of the present study may help in the prevention and treatment of atherosclerosis. Research in herbal medicine can provide an alternative solution to health problems.

*Piper sarmentosum *belongs to the family *Piperaceae*. In different parts of the world, P.s has been used traditionally to cure many diseases [17]. Phytochemically, the plant contains constituents like alkaloids (amide, flavonoids, pyrones) [18] and it has also been reported to possess pharmacological properties like antituberculosis [19] anti-cancer [20], anti-angiogenic [21], anti-diabetic [22], antimalarial [23], antioxidant [24], neuromuscular blocker [25] and antiamoebic [26]. Previous study has indicated that aqueous extract of *Piper sarmentosum *may improve endothelial function by promoting NO production in HUVECs [27] and our previous study indicated that *Ps *can be reduce atherosclerosis lesion [28]. *Piper sarmentosum has *flavonoids compounds which are widely distributed in plants which have been reported to have antioxidant, free radical scavenging abilities and anti-inflammatory effect [29]. The active extract of *P. sarmentosum *contains natural antioxidants like Naringenin (75.7%), Hesperitin (91.7%), Taxifolin/Dihydroquercitin (90.9%) and Quercetin (98.1%) which have high superoxide scavenging action [24].. The antioxidant activities of phynolic compounds like flavonoid which have anti-inflammatory action by inhibiting IL6 and TNF and inhibits the aggregation and adhesion of platelets in the blood [30]. It has been also shown that flavonoids reduce LDL oxidation, which is an important step in atherogenesis [31].

## Materials and methods

### Animals and experimental protocol

This study was approved by the Animal Ethics Committee, Universiti Kebangsaan Malaysia. Forty two male New Zealand White rabbits average body weight between 1.8 ± 2 kg were purchased from East Asia Rabbit Corporation Sdn. Bhd. Malaysia. The animals were housed separately in cages in an air-conditioned room with a 12-h light/dark cycle. All animals had free access to water. The rabbits were divided randomly into seven groups; control group (C; n = 6) was fed the rabbit chow, cholesterol group (CH; n = 6) was fed the similar diet enriched with 1% cholesterol, experimental groups (X1;n = 6, X2; n = 6, X3; n = 6 and X4; n = 6), were fed with similar diet enriched with 1% cholesterol plus different doses of water extract of *P.s *(62.5, 125, 250 and 500 mg/kg/day) respectively. The simvistatin group (Smv; n = 6) was fed with the standard diet mixed with 1% cholesterol plus simvistatin drug (1.2 mg/kg/day, Merck, NJ) [32]. The experiment was continued till 10 weeks. The blood sample was taken at the beginning of the study and the end of the study via the marginal ear vein. At the end of 10 weeks, the animals were fasted overnight and sacrificed by intravenous injection of pentobarbital (Nembutal, Abbott Laboratories, North Chicago, IL, 50 mg/kg body weight) and the abdominal aortic tissue was collected for histological studies.

### Preparation of the aqueous extract of P.s extract

The aqueous extract of *P.s *leaves were extracted in the laboratory of Furley Marketing Sdn, Bhd, Malaysia. The freeze dried powdered extract was prepared after that in the laboratory of Faculty of Pharmacy, Universiti Kebangsaan Malaysia, where the powdered extract was stored in dark bottles and kept in 4°C until used. The administration dose powder was mixed with 5 ml of water to dissolve and given to rabbits as oral dose by special needle.

### High cholesterol diet

Cholesterol powder was purchased from (Sigma Chemical Co., St. Louis, USA), it was mixed with the rabbit chow pellet (1% cholesterol, w/w, in food pellet). For each 200 g of grounded rabbit chow pellet, 2 g of cholesterol was added and mixed with a 34 ml of chloroform where cholesterol was dissolved in 99.9% chloroform and then mixed with grounded rabbit chow pellet. Chloroform was evaporated by exposing the diets as a thin layer at 50°C in oven [33].

### Diphenylpicrylhydrazyl (DPPH) for estimating antioxidant activity

1, 1-Diphenyl-2-picryl-hydrazyl (DPPH) was purchased from Sigma (USA). Ascorbic acid was purchased from Sigma USA. To measure antioxidant activity, the (DPPH), radical-scavenging assay was carried according to previous method [34,35] with slight modification. Briefly the DPPH 500 μm stock solution was prepared by adding 19.716 mg of DPPH to 100 ml of methanol and mixed until the purple colour appeared. The stock solution of vitamin C was used as positive control and sample were prepared (1000 mg/ml) with different dilution (500, 200,100, 50, 25, 12.5 and 0 mg/ml). The DPPH was mixed with the sample and vitamin C and all the tubes were left in room temperature for 30 mins in the dark. The colour was read at the absorbance at 515 nm against blank samples and the calculations of the scavenging effect (%) are as follows:

$$\text{Scavenging effect (\%)}=\left(1-\frac{A\;sample-A\;sample\;blank}{A\;control}\right)\times 100$$

A _control _= Abs of DPPH solution without sample

A_sample _= DPPH solution sample

A_sample blank_= sample without DPPH

The data is commonly reported as IC_50_, which is the concentration of antioxidant required for 50% scavenging of DPPH radicals in the specified time period

### Evaluation of Plasma Circulatory Markers (VCAM-1) and (sICAM-1)

The level concentrations of circulating Rabbit Soluble Intercellular Adhesion Molecule-1 (sICAM-1) and soluble vascular cell adhesion molecule 1(VCAM-1) were measured using *Rabbit Enzyme-Linked Immunosorbent Assay (ELISA) *and a commercially available kit (Uscn Life Science & technology company, USA) according to the manufacturer's instructions. Values of samples were calculated from a standard curve generated from standards of known concentration. Absorbance at 450 nm was determined using VERSA microplate reader (USA).

### Measurement of Plasma Rabbit High-Sensitive C - reactive protein by Enzyme-Linked Immunosorbent Assay (ELISA)

The quantification CRP level in plasma was determined using rabbit high-sensitive CRP ELISA kits from Kamiya Biomedical (Seattle, WA). The results was calibrated by the the software calculation that was provided with the VERSA microplate reader (USA) and compared to the calibration standard curve.

### Statistical analysis

Statistical analysis was carried out using the SPSS statistical package version 18 (SPSS Inc. USA). Distribution of data was examined by Kolmogrov-Smirnov test and found to be normal. Paired t-test was used to compare between 0 time and end time in the same group and ANOVA test was used to compare between the groups.

## Results

### (DPPH) for estimating antioxidant activity

**Figure 1:**
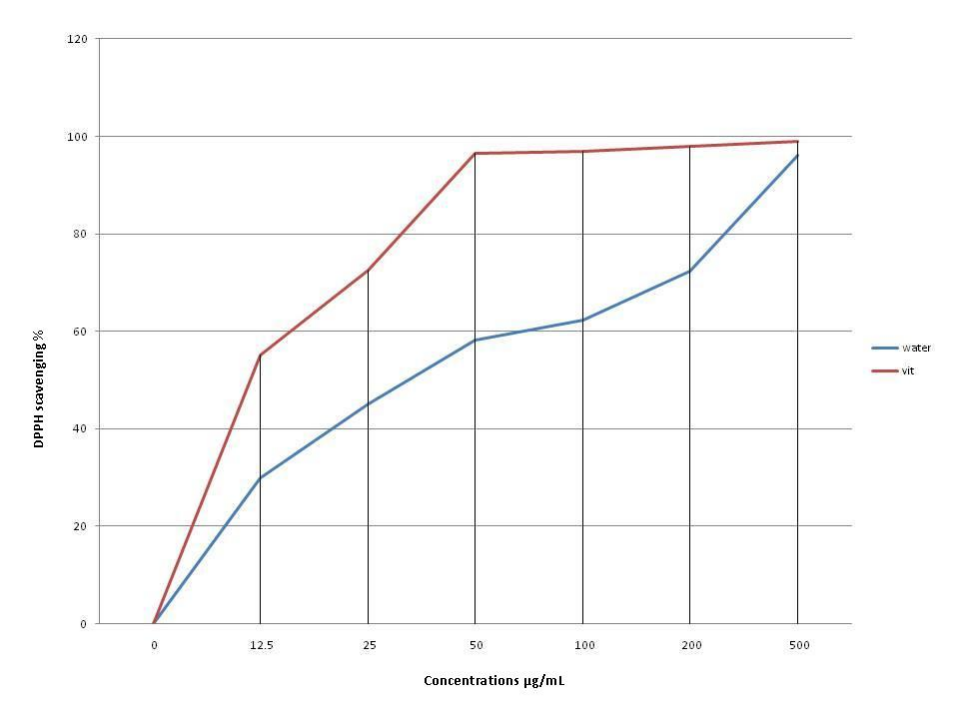
**DPPH radical scavenging activity of water extract of Ps**. Observation as IC_50_.

The result showed the DPPH radical-scavenging activity of the water extract has high antioxidant activities (Figure 1) indicated by the increase in the concentration of the sample. The antioxidant activities of the IC _50 _scavenger activity of water extract was 27.12 mg/ml.

### Plasma Circulatory Markers (VCAM-1) and (sICAM-1) and CRP

**Figure 2:**
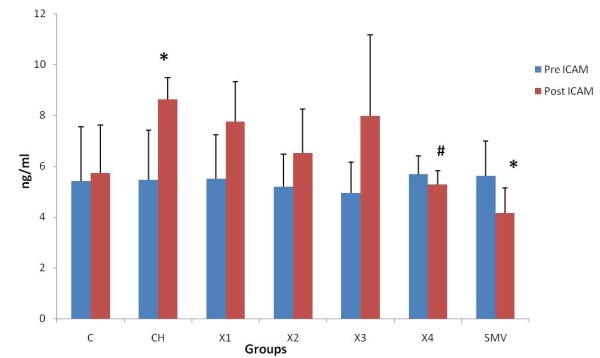
**Determination level of ICAM in the pretreatment and post treatment in the different groups**. *P < 0.05 as compare to control group. ^#^P < 0.05 as compare to atherosclerosis group.

**Figure 3:**
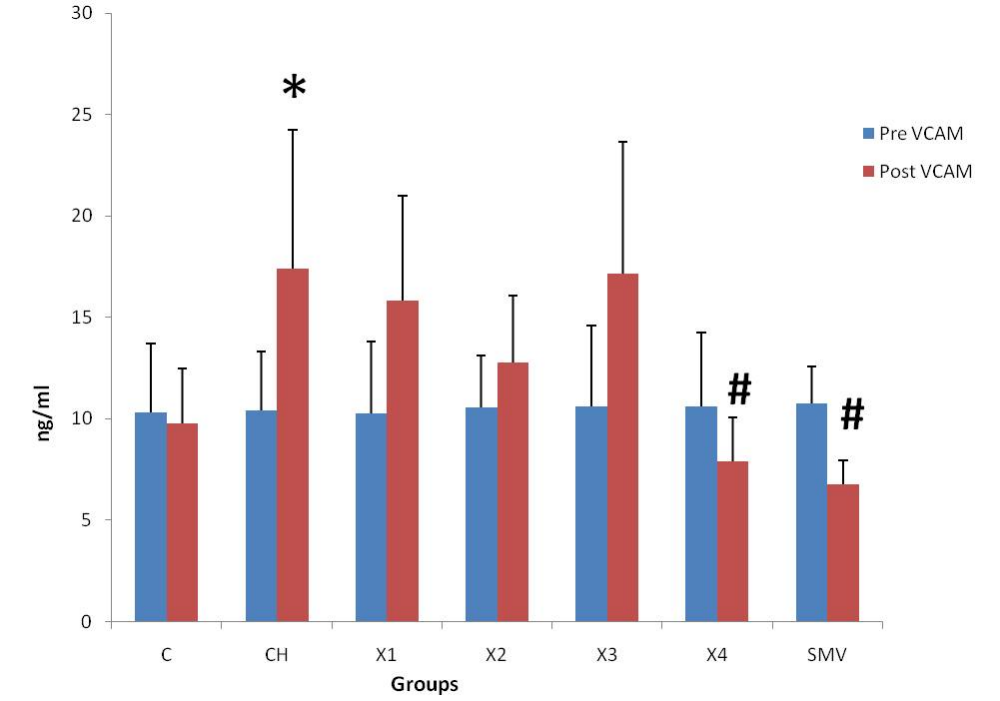
**Determination level of VCAM in the pretreatment and post treatment in the different groups**. *P < 0.05 as compare to control group.^#^P < 0.05 against atherosclerosis group.

**Figure 4:**
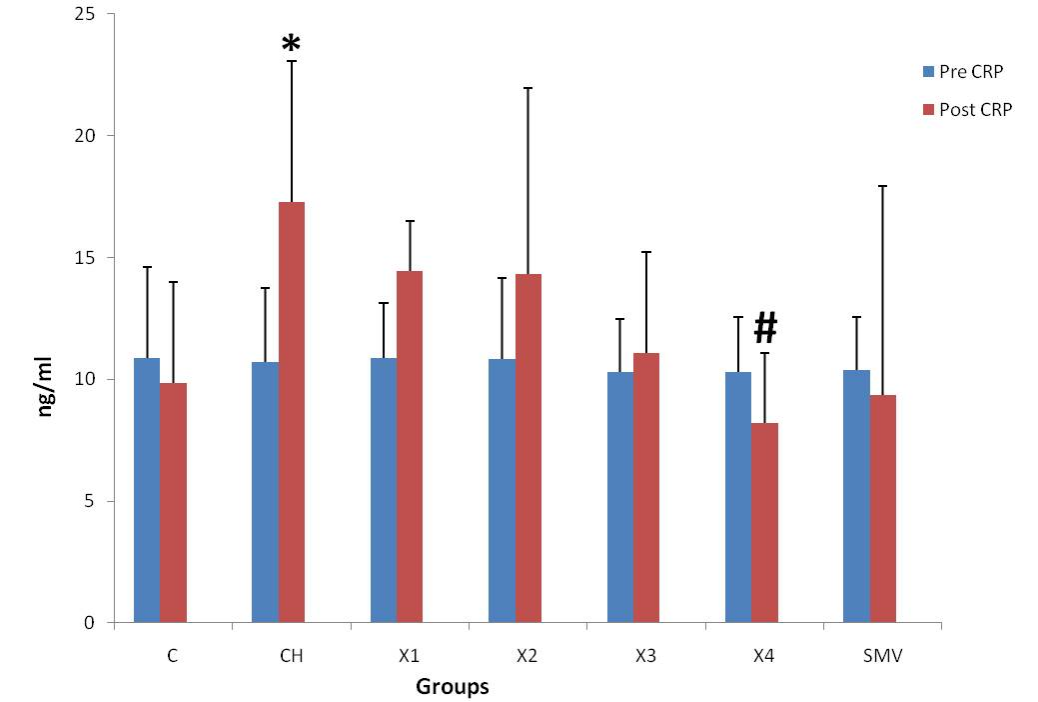
**Determination level of CRP in the pretreatment and post treatment in the different groups**. *P < 0.05 as compare to control group. ^#^P < 0.05 against atherosclerosis group.

**Figure 5:**
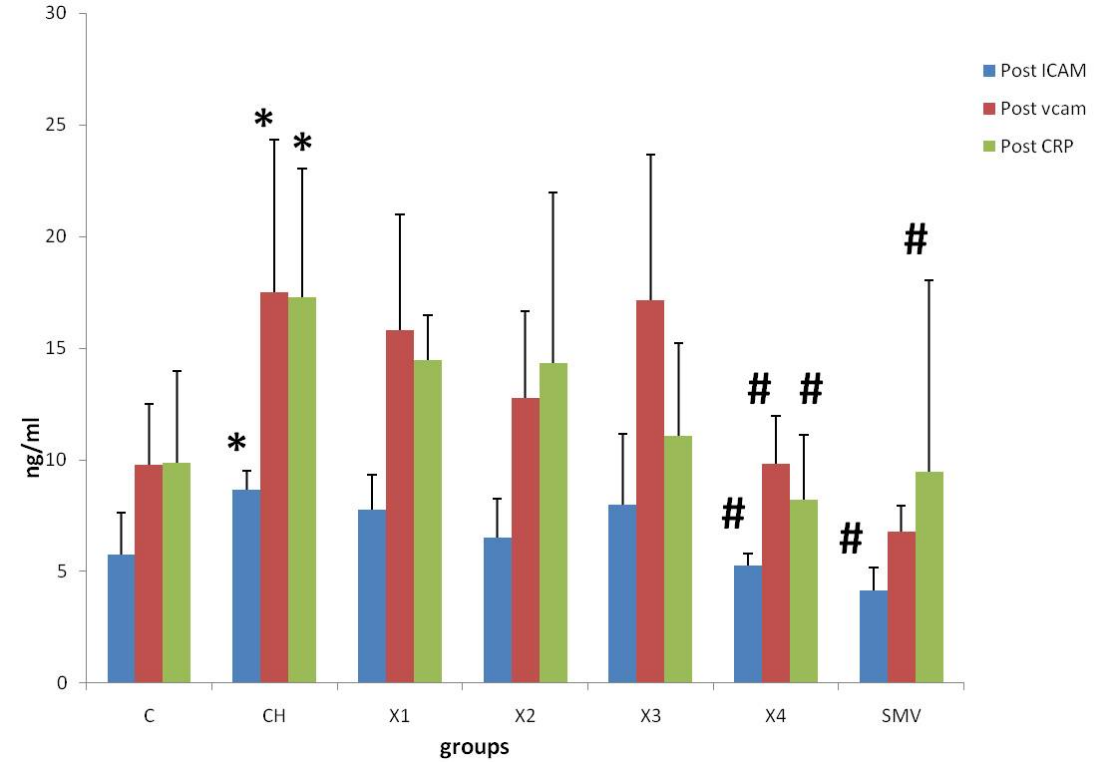
**Effect of ICAM, VCAM and CRP between the different groups**. *P < 0.05 as compare to control group. ^#^P < 0.05 against atherosclerosis group.

There was no significant difference in ICAM-1, VCAM-1 and CRP levels between 0 time and end time of experiment in the control group (C). In the atherosclerosis group there were significant increase between 0 times and end time for all ICAM-1, VCAM-1 and CRP. A similar finding was reported with the X1 group (62.5 mg/kg). In groups X2 and X3 (125 and 250 mg/kg), there were also significant differences between 0 time and end time. In groups X4 (500 mg/kg) and simvastatin, there was no significant difference between the 0 times and the end time of experiment. There was also significant increase in atherosclerotic and X1 groups as compared to control group. There were statistically significant reductions in the levels of ICAM-1, VCAM-1 and CRP at the end of treatment with water extract in group 500 mg/kg compared to atherosclerosis group. Similarly simvistatin group also showed significant reductions in the levels of ICAM-1, VCAM-1 and CRP compared to atherosclerosis group (Figure 2, 3, 4 and 5)

## Discussion

The present study showed that the water extract of P.s has high antioxidant activities and free radical-scavenging activity. The effect of antioxidants on DPPH radical scavenging may be due to their hydrogen donating ability. The antioxidant properties of *P.s *were demonstrated in previous reports [24]. In general, some antioxidants can prevent atherosclerosis by protecting LDL from oxidation and are also associated with an anti-hypercholesterolemic effect [36,37]. Several epidemiological studies have reported that increased dietary intake of natural antioxidants correlates with reduced coronary heart disease. Food rich in antioxidants plays an essential role in the prevention of cardiovascular diseases [38,39]. The extract compounds of *P.s *contains active compound like Naringenin(75.7%), Hesperitin (91.7%), Taxifolin/Dihydroquercitin(90.9%) and Quercetin (98.1%) which have high superoxide scavenging action [24]. However, there are no research reports on the effect of *P.s *on inflammatory markers in rabbits given cholesterol diet. The consumption of a cholesterol enriched diet increases the degree of lipid peroxidation, which is also one of the early processes involved in atherosclerosis.

After 10 weeks of treatment with water extract of *P.s *together with high cholesterol diet, the present study showed that ICAM, VCAM-1 and CRP levels were significantly decreased compared to rabbits given a high cholesterol diet. This study showed that treatment with this extract at the dose 500 mg/kg and simvistatin group can decrease the rise in ICAM-1, VCAM-1 and CRP. The mechanism of *P.s *in reducing the inflammatory markers is still unknown but this result suggests that this extract may have an antioxidant effect on inflammatory markers in rabbits given cholesterol diet. A previous study showed significant decreased levels of CRP in dyslipidemic patients with coronary artery disease treated with simvastatin 20 mg per day for 4 months [40]. Another study indicated that CRP-mediated inflammation is inhibited by simvastatin [39]. Previous studies showed that the elevated levels of CRP are associated with an increased risk of cardiac events [42-44]. We observed a significant increase in circulating VCAM-1, ICAM-1 levels when fed the cholesterol diet in rabbits. Increased levels of cell adhesion molecules in blood from patients with atherosclerosis have been observed in previous studies [45,46], supporting the theory that soluble inflammatory markers are involved in the pathophysiology of atherosclerosis. Prospective epidemiological studies have shown increased cardiovacsular risk associated with increased basal levels of soluble ICAM-1, VCAM-1, P-selectin, and E-selectin [47-50].

Atherosclerosis, which is a chronic inflammatory disease of arteries, is characterized by a thickening of the vascular wall and an infiltration of macrophages and lymphocytes. In animals with diet-induced or genetically determined hyperlipidemia, the earliest morphological changes in arteries include focal adherence of mononuclear leukocytes to the endothelium and accumulation of monocyte-derived foam cells in the intima [51,52].

The mechanisms that link diet-induced hyperlipidemia in the evolution of cellular changes in the arterial wall still remain poorly understood. VCAM-1 expression might conceivably result from macrophage accumulation because activated macrophages can produce many of the cytokines known to stimulate endothelial VCAM-1 expression [53]. Oxidative stress induced inflammatory responses cause damage to the vasculature and may play an important role in the development of many diseases including atherosclerosis [54]. Activation of endothelial cells by oxidants may lead to a wide range of functional changes such as an increased expression of VCAM-1, ICAM-1, and E-selectin, and the production of chemokines, such as monocyte chemoattractant peptide-1. The resulting attraction and transendothelial migration of monocytes are believed to be critical to the initiation and progression of atherosclerosis [55]. The protective effect of antioxidant is still not clear but it may serve as a protective function by preventing the oxidation of LDL.

## Conclusion

This study suggests that hypercholesterolemic atherosclerosis is associated with an increase in inflammatory markers and that *Piper sarmentosum *is effective in reducing the inflammation which is important in the process of atherosclerosis.

## Abbreviations

(*P.s*): Piper *sarmentosum*; (C): control group; (CH): Atherogenic group; (x1): 1%cholesterol together with water extracts of P.S with doses 62.5 mg/kg group; (X2): 1% cholesterol together with water extracts of P.S with doses 125 mg/kg;(X3): 1% cholesterol together with water extracts of P.S with doses 250 mg/kg;(X4): 1% cholesterol together with water extracts of P.S with doses 500 mg/kg;(Smv): 1% cholesterol supplemented with simvistatin drug 1.2 mg/kg; (HO-1):heme oxygenase-1; LDL: Low Density Lipoprotein; CVD: Cardiovascular D(VCAM-1): vascular cell adhesion molecule-1;(ICAM-1): intercellular adhesion molecule-1;(CRP);(CAMs): cellular adhesion molecule-1;(DPPH): 1,1-Diphenyl-2-picryl-hydrazyl.

## Competing interests

The authors declare that they have no competing interests.

## Authors' contributions

ZZ was involved in supervising the project, and revising the manuscript critically for important intellectual content. AA carried out all aspects of experiments, design and data analysis, and drafted the manuscript and revising it critically for important intellectual content. FO was involved in interpreting the results and revising it critically for important intellectual content. SD was involved in histological interpretation of results, design, grammars, technical assistance in preparing the manuscript. Hm for statistical analysis and design and NMN were involved in the extraction of *Piper sarmentosum *and revising the manuscript critically for important intellectual content. All authors have read and approved the final manuscript.
